# Hypoxia-Induced Exosomal miR-1225-5p Accelerates Colorectal Cancer Progression by Targeting Carboxypeptidase M

**DOI:** 10.14740/wjon2766

**Published:** 2026-06-25

**Authors:** Yue Hui Guo, Qing Yun Zhu, Shi Wei Chen, Yan Xiang Li, Chuan Chen, Cai Fang Ni

**Affiliations:** a Department of Interventional Radiology, the First Affiliated Hospital of Soochow University, Suzhou 215006, Jiangsu, China; b Department of Interventional Radiology, Gongli Hospital of Shanghai Pudong New Area, Shanghai 200135, China

**Keywords:** Colorectal cancer, Hypoxia, miR-1225-5p, *CPM*, Exosomes, Tumor progression

## Abstract

**Background:**

Colorectal cancer (CRC) remains a considerable public health burden worldwide, with hypoxia emerging as a key driver of tumor aggressiveness. Within the hypoxic tumor microenvironment (TME), exosomes function as vital vehicles for intercellular communication, especially through their cargo of microRNAs (miRNAs). Despite the growing recognition of this phenomenon, the specific functions of hypoxia-induced exosomal miRNAs in CRC remain inadequately defined.

**Methods:**

Human CRC cell lines (SW620 and HCT116) were subjected to normoxic or hypoxic conditions. Exosomes were isolated via ultracentrifugation and rigorously characterized by transmission electron microscopy (TEM) and dynamic light scattering (DLS). Quantitative real-time PCR (qRT-PCR) profiling identified miR-1225-5p as significantly enriched under hypoxia. The uptake of Cy3-labeled miR-1225-5p-loaded and PKH67-labeled exosomes by CRC cells was confirmed. The impact of these phenomena on cell progression capabilities was subsequently evaluated through the implementation of cell counting kit-8, colony formation, Transwell invasion, and wound-healing assays. The validation of the miR-1225-5p-carboxypeptidase M (*CPM*) regulatory axis was conducted through a series of rigorous methods, including luciferase reporter assays, Western blot analysis, and siRNA-mediated knockdown. Bioinformatic assessments via UALCAN examined correlations between *CPM* expression and clinical CRC prognosis.

**Results:**

Hypoxic CRC cell-derived exosomes exhibited markedly increased miR-1225-5p content. These exosomes effectively transferred miR-1225-5p to recipient CRC cells, thereby enhancing their malignant behaviors. Mechanistically, miR-1225-5p directly suppressed *CPM* expression by interacting with its 3'UTR. Bioinformatics analysis demonstrated that lower CPM expression correlates with poor patient prognosis, indicating a tumor-suppressive role. The downregulation of *CPM* independently recapitulated the oncogenic phenotypes induced by miR-1225-5p, whereas the inhibition of miR-1225-5p reversed these effects, suppressing CRC cell malignancy.

**Conclusion:**

The present study identifies a novel hypoxia-driven exosome-mediated miRNA pathway, whereby miR-1225-5p promotes CRC progression through targeted inhibition of the tumor suppressor gene, *CPM*. These findings contribute to our expanded understanding of exosomal RNA signaling within a hypoxic TME, thus identifying potential therapeutic targets.

## Introduction

Colorectal cancer (CRC) continues to impose a serious global health burden, posing a major challenge to medical communities worldwide with persistently high incidence and mortality rates, responsible for approximately one-tenth of all cancer diagnoses and deaths each year [1–3]. Despite the advancement in early detection methods and therapeutic interventions, which have led to improvements in survival rates among patients in the early stages of the disease, the prognosis for those with advanced CRC remains poor [2, 4]. This is largely due to tumor recurrence, distant metastases, and increasing resistance to conventional treatments [2, 3, 5, 6]. Addressing this pressing issue necessitates a deeper exploration of the intricate molecular networks that drive CRC progression, aiming to reveal novel therapeutic targets and ultimately enhance patient survival outcomes.

Exosomes are nanoscale vesicles released by diverse cell populations and are increasingly recognized for their critical functions in mediating intercellular communication within the tumor microenvironment (TME) [7, 8]. They facilitate the transfer of diverse bioactive molecules, including proteins, lipids, DNA, and notably, non-coding RNAs [9–11]. MicroRNAs (miRNAs) are small, non-coding RNA molecules that critically participate in the intricate regulation of gene expression at the post-transcriptional level [12, 13] and profoundly shape cancer biology by modulating cellular processes like proliferation, apoptosis, metastasis, and immune evasion [14–19]. A body of recent evidence has suggested that exosome-mediated transfer of miRNAs has a powerful impact on cancer progression, particularly by altering the TME [20, 21].

Hypoxia, a hallmark of solid tumors, exerts a dominant influence on the TME, affecting exosome secretion and altering miRNA expression profiles within these vesicles [22–24]. In the context of hypoxic conditions, cancer cells frequently augment exosome production and selectively package specific miRNAs, thereby enhancing intercellular communication and facilitating tumor progression, invasion, and metastasis [23–25]. Mechanistically, these hypoxia-driven cellular adaptations are primarily orchestrated by hypoxia-inducible factor 1-alpha (HIF-1α). As a master transcription factor, HIF-1α directly binds to the promoter regions of its target genes to transcriptionally regulate the expression of specific miRNAs, which are subsequently sorted into exosomes to facilitate tumor progression.

Among these molecules, exosomal miR-1225-5p has attracted considerable attention due to its role in modulating tumor cell growth and metastasis in cancers such as ovarian and gastric cancer [26, 27]. Nevertheless, the precise functions and regulatory pathways of exosome-derived miR-1225-5p in CRC remain largely unexplored, emphasizing the necessity for further detailed investigations.

Carboxypeptidase M (CPM), a membrane-anchored zinc metalloprotease, has increasingly emerged as a crucial molecule in cancer research due to its multifaceted roles in tumorigenesis and malignant progression. Initially, CPM garnered significant oncological attention as a highly sensitive and specific diagnostic biomarker. Genomic and histopathological studies have consistently demonstrated that CPM is frequently co-amplified with MDM2 and CDK4 within the chromosome 12q13-15 amplicon, a molecular signature particularly characteristic of well-differentiated and dedifferentiated liposarcomas (WDLPS/DDLPS) [28–31]. Beyond its established diagnostic utility in soft tissue sarcomas, recent advancements have elucidated the active functional contributions of CPM in driving tumor aggressiveness across a broader spectrum of cancers [32, 33]. In some cancers, CPM exhibits oncogenic properties, whereas in CRC, it functions as a tumor suppressor [32, 33]; however, the precise molecular networks controlling its expression and its downstream mechanisms remain largely unknown. Mechanistically, CPM facilitates these malignant phenotypes by enzymatically modulating the local peptide microenvironment, such as generating active agonists for the kinin B1 receptor, and through dynamic molecular crosstalk with critical oncogenic networks, including the epidermal growth factor receptor (EGFR) signaling pathway. Collectively, these progressive findings highlight that CPM acts not merely as a passive diagnostic marker, but as an active driver of tumor progression, positioning it as a promising prognostic indicator and a potential therapeutic target in oncology. Despite the emerging recognition of its clinical relevance, the molecular mechanisms controlling *CPM* expression in CRC remain largely unknown, highlighting a critical gap in our understanding that demands further exploration.

In this study, we aimed to investigate the role of hypoxia-induced exosomal miR-1225-5p in CRC progression and elucidate the underlying molecular mechanisms mediated by its target gene, *CPM*. The results highlighted that hypoxia promotes the secretion of exosomes-enriched miR-1225-5p, which are transferred to recipient CRC cells to enhance proliferation, migration, and invasion by directly targeting *CPM*. These findings offer unique perspectives on the intercellular communication facilitated by exosomal miRNAs in CRC and underscore the potential of the miR-1225-5p/*CPM* axis as a therapy candidate.

## Materials and Methods

### Cell culture and siRNA/miRNA transfection

Human CRC cell lines HCT116 and SW620, normal colorectal cell CCD-18Co, as well as the human embryonic kidney cell line 293T, were obtained from the Cell Bank of the Chinese Academy of Sciences (Shanghai, China). HCT116, SW620 cells, and CCD-18Co cells were maintained at 37 °C in a humidified incubator with 5% CO_2_, using RPMI-1640 medium (Gibco, 11875-093) supplemented with 10% fetal bovine serum (Gibco, 10099-141), 100 U/mL penicillin, and 100 µg/mL streptomycin (Solarbio, P1400). The 293T cells were maintained in high-glucose DMEM (Gibco, 11965-092) with the same supplements under identical incubation conditions. For hypoxia treatment, cells were cultured in a modular hypoxia incubator chamber (STEMCELL Technologies, 27310) filled with a gas mixture containing 1% O_2_, 5% CO_2_, and 94% N_2_ for 24 h. Normoxic cells were maintained at 21% O_2_.

For small interfering RNA (siRNA) transfection experiments, HCT116 or SW620 cells were first seeded into six-well culture plates and incubated under appropriate conditions until they achieved approximately 60% confluency. Subsequently, the cells were transfected with either *HIF-1α* siRNA (5'-CUGAUGACCAGCAACUUGATT-3', GenePharma, China), or *CPM* siRNA (si-*CPM*-1: 5'-ACUAUUGAUCAGAUUUGUGAU-3'; si-*CPM*-2: 5'-UUUUGACGGCUUCAAAUCCAU-3', GenePharma, China), or non-targeting scrambled control siRNA (si-NC, 5'-CGAACUCACUGGUCUGACC-3', GenePharma, China). This transfection process utilized Lipofectamine RNAiMAX transfection reagent (supplied by Invitrogen, a brand under Life Technologies) and was performed following the protocols provided by the manufacturer. The efficacy of the transfection process was subsequently validated by quantitative real-time PCR (qRT-PCR) to analyze the levels of miR-1225-5p or *HIF-1α* and *CPM* mRNA. Each experiment was performed in at least three independent biological replicates.

### Exosome isolation and characterization

Exosomes were isolated from cell culture supernatants collected after 48 h of incubation in exosome-depleted medium, which was prepared by ultracentrifuging FBS at 120,000 × g for 720 min. To eliminate cells and debris, the conditioned media (CM) were sequentially centrifuged at 300 × g for 10 min, 2,000 × g for 20 min, and 10,000 × g for 30 min. The resulting supernatants were subjected to ultracentrifugation at 120,000 × g for 70 min at 4 °C using an Optima XPN-100 Ultracentrifuge (Beckman Coulter, USA) to collect the exosomal pellet. The pellets were then washed with phosphate-buffered saline (PBS; Gibco, 10010-023) and ultracentrifuged again under identical conditions to ensure purity. These differential ultracentrifugation speeds, durations, and washing steps were performed in strict adherence to the Minimal Information for Studies of Extracellular Vesicles 2018 (MISEV2018) guidelines.

Exosome morphology was visualized by transmission electron microscopy (TEM; HT7700, Hitachi, Japan) after fixation with 2% paraformaldehyde (Servicebio, G1101). Particle size and distribution were determined by dynamic light scattering (DLS) using the Zetasizer Nano ZS90 (Malvern Panalytical, UK). Furthermore, exosome concentration and exact size distribution profiles were quantified by nanoparticle tracking analysis (NTA) using a Nanosight NS300 instrument (Malvern Panalytical, UK). Samples were diluted in PBS to achieve an optimal particle concentration (10^7^–10^9^ particles/mL) for analysis. Exosome-containing conditioned medium (Exo CM): exosomes isolated by ultracentrifugation and resuspended in serum-free medium; Exosome-depleted conditioned medium (Exo-depleted CM): supernatant after exosomes depletion by ultracentrifugation. Exosome doses in functional assays were normalized to the same total protein concentration measured using a BCA assay kit (Beyotime, P0010S).

### Exosome labeling and uptake assay

The evaluation of exosomes uptake was conducted by labeling purified exosome samples with the PKH67 Green Fluorescent Cell Linker Kit (Sigma-Aldrich, MINI67). In summary, 20–40 µg of purified exosome samples was resuspended in 100 µL PBS and incubated with 2 µL PKH67 dye (10 µM stock solution) for 5 min at room temperature. To terminate the labeling reaction, 1% BSA was added to neutralize unbound dye. To minimize dye aggregation artifacts, labeled exosomes were washed with PBS and subjected to ultracentrifugation at 100,000 × g for 70 min to re-isolate the labeled exosomes and remove unincorporated dye. A dye-only control (PKH67 incubated with PBS under identical conditions) was included to assess background fluorescence. The resuspended exosomes were then incubated with HCT116 cells, which had been grown on glass coverslips, for either 1 or 6 h at 37 °C. Cells were subsequently rinsed with PBS, fixed using 4% paraformaldehyde (Servicebio, G1101), and counterstained with DAPI (Beyotime, C1006) to visualize nuclei. The internalization of PKH67-labeled exosomes was then observed using a confocal laser scanning microscope (Leica TCS SP8, Germany).

### RNA extraction and qRT-PCR

Total RNA was extracted from cells using TRIzol Reagent (Invitrogen, 15596018), and from exosome samples using the exoRNeasy Serum/Plasma Midi Kit (Qiagen, 77044). For exosomal RNA, cel-miR-39 (Qiagen) was spiked in as an exogenous control during extraction to monitor efficiency. For the analysis of miRNA, reverse transcription was performed using the miR-X RNA First-Strand Synthesis Kit (Takara, 638313). Quantification by real-time PCR was then conducted using TB Green Premix Ex Taq II (Takara, RR820A) on a QuantStudio 5 Real-Time PCR System (Applied Biosystems, USA). For mRNA detection, reverse transcription was carried out using the PrimeScript RT Reagent Kit with gDNA Eraser (Takara, RR047A, Japan), followed by qRT-PCR under previously described conditions. Relative expression levels were calculated using the 2^-ΔΔCt^ method. The detailed sequences of all primers used in this study, along with their optimized annealing temperatures, are summarized in Supplementary Material 1 (wjon.elmerpub.com).

### Immunofluorescence (IF)

To visualize the intracellular uptake of exosomal miR-1225-5p, Cy3-labeled miR-1225-5p was transfected into SW620 or HCT116 donor cells. Following a 48-h transfection period, the CM was collected and the isolation of the exosomes was conducted using ultracentrifugation as previously described. The purified exosome samples containing Cy3-labeled miR-1225-5p were then incubated with recipient HCT116 cells at a concentration of approximately 20 µg/mL for a period of 6 h. Following this, the cells were washed thrice with cold PBS. The cells were fixed with 4% paraformaldehyde for 20 min at room temperature, followed by permeabilization with 0.1% Triton X-100 (Solarbio, T8200) for 10 min. After counterstaining with DAPI for 5 min in the dark, coverslips were mounted using anti-fade mounting medium (Beyotime, P0126) on slides. Images were captured using a confocal laser scanning microscope (Leica TCS SP8, Germany). Red fluorescence (Cy3) was used to indicate the presence of exosomal miR-1225-5p, and blue fluorescence (DAPI) marked the nuclei.

### RNase protection assay for exosomal miRNAs

In order to ascertain the location of miR-1225-5p encapsulated within the vesicular lumen of the exosomes, rather than in the extracellular environment, an RNase protection assay was conducted. CM from CRC cells were allocated to three treatment groups: untreated (control), RNase A alone, or RNase A combined with Triton X-100. RNase A (Thermo Fisher, EN0531) was utilized at a final concentration of 2 µg/mL, and treatment was carried out at 37 °C for 30 min. In the permeabilization group, 0.1% Triton X-100 was added to disrupt the exosomal membrane, thereby allowing RNase access to the internal contents. Subsequent to the administration of treatment, total RNA was extracted from each group, and the expression levels of miR-1225-5p were then quantified. The results were subsequently compared across different experimental conditions.

### Cell proliferation assay (CCK-8)

Cell proliferation was evaluated using the cell counting kit-8 (CCK-8, Dojindo, CK04-11). SW620 and HCT116 cells were plated in 96-well plates (Corning, 3599) at a density of 2,000 cells per well in 100 µL of complete culture medium. After overnight attachment, cells were treated with PBS (negative control), miR-NC exosomes, miR-1225-5p exosomes, inhibitor-NC exosomes, or miR-1225-5p inhibitor exosomes (final concentration: 20 μg/mL). At selected time intervals (12 to 72 h), 10 µL of CCK-8 reagent was added to each well and incubated for 2 h at 37 °C in the dark. Absorbance at 450 nm was then recorded using a microplate reader (SpectraMax i3x, Molecular Devices, USA). This parameter is directly proportional to the number of viable cells. Subsequent to this, the background value of the media-only wells was deducted from each reading. Each experimental condition was performed with six technical replicates and repeated in three independent biological experiments.

### Cell invasion assay (Transwell)

Cell invasion was assessed using 24-well Transwell chambers (Corning, 3422) equipped with polycarbonate membranes (8-µm pore size) that had been pre-coated with Matrigel (Corning, 354234) to simulate the extracellular matrix. Prior to the Transwell assay, the cells were pretreated with serum-free medium containing 20 µg/mL exosomes (or the corresponding control) for 6 h. HCT116 or SW620 cells (5 × 10^4^ per insert) suspended in 200 µL of serum-free RPMI-1640 medium and were seeded into the upper chambers. Simultaneously, 600 µL of complete medium supplemented with 10% FBS was placed in the lower chamber to act as a chemoattractant. Following a 48 h incubation at 37 °C, cells remaining on the upper side of the membrane were carefully removed using a cotton swab. Invaded cells on the underside were then fixed with 4% paraformaldehyde for 20 min and stained with 0.1% crystal violet (Solarbio, G1063) for 15 min. The number of invaded cells was quantified under a light microscope (Olympus CKX53, Japan) by counting five random fields per insert. Experiments were performed in triplicate.

### Colony formation assay

SW620 or HCT116 cells were seeded into six-well plates (Corning, 3516) at a density of 500–800 cells per well, depending on the cell line growth rate, and allowed to adhere overnight. Cells were cultured in complete medium containing 20 µg/mL exosomes (or the corresponding control) for 14 days, with medium refreshed every 2–3 days. When visible colonies (> 50 cells) appeared, wells were gently washed with PBS, fixed in 4% paraformaldehyde for 30 min at room temperature, and stained with 0.1% crystal violet for 15–20 min. Residual dye was removed by rinsing with distilled water, and plates were subsequently air-dried. At least five random fields per well were examined under an inverted microscope (Olympus CKX53, Japan), and colony numbers were counted manually. Each condition was tested in triplicate wells.

### Wound healing assay

HCT116 or SW620 cells were seeded in six-well plates (Corning, 3516) at a density of 5 × 10^5^ cells per well and cultured until they formed a confluent monolayer. Prior to the wound healing assay, the cells were pretreated with serum-free medium containing 20 µg/mL exosomes (or the corresponding control) for 6 h. A sterile 200 µL pipette tip (Axygen, T-200-Y) was used to create a straight scratch (wound) across the cell monolayer. The detached cells were then gently removed by washing twice with PBS, and the medium was replaced with serum-free RPMI-1640 to minimize proliferation. To further exclude proliferation effects, parallel experiments were performed with 10 µg/mL mitomycin C pretreatment for 2 h prior to wounding. Wound areas were imaged at 0 h and after 24 or 48 h of incubation using an Olympus CKX53 inverted microscope (Japan) fitted with a DP22 digital camera. The wound width was measured using ImageJ software (NIH, USA). Each group was tested in triplicate, and at least five random fields were analyzed per condition.

### Bioinformatic analysis using the University of Alabama at Birmingham Cancer data analysis portal (UALCAN) database

The mRNA expression levels of CPM and its clinical prognostic significance in CRC were comprehensively evaluated using the UALCAN online portal [34], a comprehensive interactive web resource for analyzing cancer transcriptome data from The Cancer Genome Atlas (TCGA). First, the differential expression of CPM was compared between primary CRC tumor tissues and normal adjacent controls. To further investigate the association between CPM expression and clinicopathological features, the data were stratified and analyzed across various subgroups, including tumor pathological stages (stages I–IV), patient demographics (race and age), and distinct histological subtypes (adenocarcinoma and mucinous adenocarcinoma). Finally, the prognostic value of CPM was assessed through survival analysis generated by the UALCAN platform, which evaluated the correlation between CPM expression levels and the overall survival of CRC patients, with the statistical significance (P < 0.05) determined by the built-in log-rank test. In the UALCAN database, patients were stratified into two groups based on the upper quartile (75th percentile) of gene expression. Specifically, patients with expression levels in the top 25% were defined as the high expression group, while the remaining 75% were categorized as the low/medium expression group.

### Chromatin immunoprecipitation (ChIP)

We employed a ChIP assay kit to confirm the enrichment level of HIF-1α in the promoter region of miR-1225-5p. Cells were fixed using 1% formaldehyde for 10 min, followed by ultrasonic treatment to randomly shear genomic DNA into fragments ranging from 200 to 800 bp. Subsequently, an HIF-1α-specific antibody (Cell Signaling Technology, #36169) was used to perform immunoprecipitation on the DNA fragments. After that, 100 µL of distilled water was utilized for the purification and elution of ChIP-derived DNA, and 2.5 µL of this ChIP-DNA was subjected to detection via ChIP-qPCR. IgG was set as control. Diverse primer pairs were applied to determine the enrichment of HIF-1α in the miR-1225-5p promoter region.

### Western blotting (WB)

Cells were lysed in RIPA buffer (Beyotime, P0013B) supplemented with protease and phosphatase inhibitors (Thermo Scientific, 78440) to extract total protein. Protein concentrations were quantified using a BCA assay kit (Beyotime, P0010S). Equivalent protein amounts (20–30 µg) were resolved on 10% SDS-PAGE gels and transferred to PVDF membranes (Millipore, IPVH00010). Membranes were blocked in 5% non-fat milk and incubated overnight at 4 °C with the appropriate primary antibodies: anti-CD63 (Abcam, ab216130, 1:1,000), anti-Alix (Cell Signaling Technology, CST #2171, 1:1,000), anti-HSP70 (Proteintech, 10654-1-AP, 1:1,000), anti-calnexin (Abcam, ab22595, 1:1,000), anti-CPM (Abcam, ab150405, 1:1,000), and anti-GAPDH (Proteintech, 60004-1-Ig, 1:5,000). Following a thorough washing step, the membranes were incubated with horseradish peroxidase (HRP)-conjugated secondary antibodies (Proteintech, SA00001-1 and SA00001-2, 1:5,000) for a duration of 1 h at room temperature. Protein bands were visualized using ECL detection reagent (Thermo Fisher, 32106) and imaged with a chemiluminescence system (Bio-Rad ChemiDoc XRS+, USA). Baseline expression of CPM protein in HCT116 and SW620 cell lysates was confirmed by WB (data not shown).

### Plasmid construction and dual-luciferase reporter assay

The 3'UTR of human CPM, including the putative binding site for miR-1225-5p, was inserted downstream of the Renilla luciferase gene in the psiCHECK-2 reporter vector (Promega, C8021). A mutant construct was then generated by site-directed mutagenesis using the Mut Express II Fast Mutagenesis Kit (Vazyme, C214-01). The 293T and HCT116 cells were co-transfected with WT or MT constructs along with miR-1225-5p mimic, inhibitor, or their respective negative controls using Lipofectamine 3000 (Thermo Fisher, L3000008). Following a 48-h period, the luciferase activity was measured using the Dual-Luciferase^®^ Reporter Assay System (Promega, E1910) on a luminometer (Berthold Centro XS3 LB960, Germany). The normalization of the data was facilitated by the use of firefly luciferase activity.

### Inhibition of exosome secretion

To inhibit exosome secretion, cells were treated with 10 µM GW4869 (Sigma-Aldrich, D1692) or vehicle control (DMSO) for 24 h. Cell viability was assessed by CCK-8 assay to ensure no significant cytotoxicity at this concentration. The reduction in exosomes release was confirmed by NTA analysis of CM. An alternative approach using shRNA targeting neutral sphingomyelinase 2 (nSMase2) (sh-nSMase2) was also tested in parallel experiments to confirm the specificity of GW4869 effects. The knockdown efficiency of nSMase2 was confirmed by qRT-PCR prior to functional assays.

### Lentivirus generation and stable cell line construction

To establish the stable CPM knockdown cell line for *in vivo* experiments, recombinant lentiviral vectors encoding short hairpin RNA (shRNA) targeting CPM (sh-CPM) and a non-targeting scrambled control (sh-NC) were constructed and packaged by GenePharma (Shanghai, China). The lentiviral backbone utilized was a puromycin-resistant vector (pLKO.1-puro). SW620 cells were seeded into six-well plates (Corning) and cultured until reaching 40–50% confluence. For viral transduction, the cells were incubated with the lentiviral particles at a multiplicity of infection (MOI) of 20 in the presence of 8 µg/mL Polybrene (Sigma-Aldrich, TR-1003) to enhance infection efficiency. After 24 h of co-incubation at 37 °C, the virus-containing medium was gently aspirated and replaced with fresh complete RPMI-1640 medium. At 48 h post-transduction, the cells were subjected to antibiotic selection by adding 2 µg/mL puromycin (Sigma-Aldrich, P8833). The puromycin-containing medium was refreshed every 2–3 days for approximately 10–14 days to eliminate untransfected cells and establish stable cell pools. The stable knockdown efficiency of CPM in the surviving SW620 cells was subsequently validated at both the mRNA and protein levels via qRT-PCR and WB, respectively, prior to subcutaneous implantation in mice.

### Tumor xenograft experiment

Male and female BALB/c nude mice (five animals per group) were subcutaneously implanted with SW620 cells. Once tumor volumes reached approximately 100 mm^3^, the mice were randomly assigned to three groups: Exo: the tumors were implanted with normal SW620 cells and injected with exosomes from hypoxia-exposed SW620 cells; miR-1225-5p inhibitor Exo: the tumors were implanted with normal SW620 cells and injected with exosomes from hypoxia-exposed SW620 cells which were previously transfected with a miR-1225-5p inhibitor; miR-1225-5p inhibitor Exo+sh-CPM: the tumors were implanted with SW620 cells pretreated with sh-CPM and injected with exosomes from hypoxia-exposed SW620 cells co-transfected with the miR-1225-5p inhibitor. Mice were randomly allocated to experimental groups, and the investigator measuring tumor volumes was blinded to the group allocations. Each group received intratumoral administration of 10 µg exosomes isolated from hypoxia-exposed SW620 cells (these cells were either left untreated or transfected with a miR-1225-5p inhibitor) twice a week. On the 40th day, the mice were euthanized via rapid cervical dislocation, and the tumors were dissected and weighed.

Subsequently, the tumor tissues were subjected to immunohistochemical (IHC) staining. Paraffin-embedded sections were deparaffinized using xylene and rehydrated via a graded series of 100% down to 70% ethanol (Sigma-Aldrich). Antigen retrieval was achieved by microwave heating the sections in citrate buffer (pH 6.0, Sigma-Aldrich). Endogenous peroxidase activity was quenched with 3% hydrogen peroxide (Sigma-Aldrich) for 10 min at room temperature (RT), followed by a 30-min blocking step with 5% serum (Sigma-Aldrich) at RT. Primary antibodies targeting CPM, Ki-67, and PCNA (Sigma-Aldrich), diluted in blocking solution, were incubated with the sections overnight at 4 °C in a humidified environment. After thorough washing, the sections were incubated with HRP-conjugated secondary antibodies (Sigma-Aldrich) for 1 h at RT, and HRP activity was detected using a DAB (3,3'-diaminobenzidine) substrate (Thermo Fisher Scientific). The sections were counterstained with hematoxylin to visualize cell nuclei, then dehydrated through a graded ethanol series, cleared in xylene, and mounted with coverslips. Stained sections were observed and imaged under an Olympus light microscope.

The animal experiment was approved by the Animal Ethics Committee of the First Affiliated Hospital of Soochow University. This study was conducted in compliance with all the applicable institutional ethical guidelines for the care, welfare, and use of animals.

### Statistical analysis

Data are presented as mean ± standard deviation (SD) from at least three independent experiments. Statistical comparisons were conducted using GraphPad Prism 9.0 (GraphPad Software, USA). Differences between two groups were evaluated by Student’s *t*-test, while multiple group comparisons were assessed using one-way analysis of variance (ANOVA) followed by Tukey’s multiple comparison test. A P-value < 0.05 was considered statistically significant.

## Results

### Hypoxia promotes exosome secretion from CRC cells

**Figure 1:**
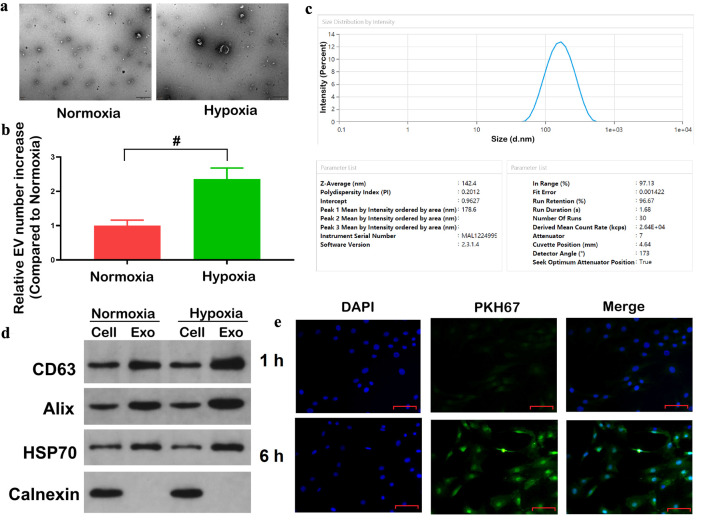
Hypoxia promotes exosome secretion from colorectal cancer cells. (a) TEM of exosomes derived from HCT116 cells under normoxic and hypoxic conditions. Scale bar = 500 nm. (b) Quantification of relative exosomes numbers under normoxia and hypoxia by NTA. (c) Size distribution and mean diameter of isolated exosomes determined by DLS. (d) The protein levels of exosomal markers (CD63, Alix, HSP70) and negative control Calnexin in cell lysates and exosomal fractions. (e) The co-culture of PKH67-labelled exosome samples derived from hypoxic HCT116 cells with HCT116 cells was conducted over a period of 1 and 6 h. Scale bar = 50 µm. Confocal microscopy showed internalization of exosomes (green) into DAPI-stained cells (blue). Data are presented as mean ± SD (n = 3 independent experiments). ^#^P < 0.05 between normoxic and hypoxia groups. TEM: transmission electron microscopy; NTA: nanoparticle tracking analysis; DLS: dynamic light scattering; DAPI: 4',6-diamidino-2-phenylindole.

To investigate whether hypoxic conditions influence the exosome secretion in CRC cells, we cultured HCT116 cells under normoxic or hypoxic conditions and isolated exosomes from the culture supernatants. TEM revealed the presence of cup-shaped exosomes exhibiting the characteristic morphology of exosomes in both experimental conditions, but the abundance of exosomes was significantly increased under hypoxia (Fig. 1a). Furthermore, NTA was utilized to quantify particle concentration and revealed a substantial augmentation in exosome production under hypoxic conditions (Fig. 1b, P < 0.05).

The DLS analysis showed a size range of 80–200 nm, accompanied by a Z-average diameter of 142.4 nm, a measurement consistent with those exhibited by typical exosome samples (Fig. 1c). WB confirmed the presence of established exosomal markers (CD63, Alix, and HSP70) in the exosome fractions, whereas calnexin, an endoplasmic reticulum protein, was undetectable, supporting the specificity and purity of the isolated exosomes (Fig. 1d). Therefore, to assess whether exosomes secreted under hypoxia could be internalized by other CRC cells, PKH67-labelled exosomes derived from hypoxic HCT116 cells were co-incubated with normoxic HCT116 cells. Confocal microscopy observations revealed that uptake of green fluorescent PKH67-labeled exosomes with DAPI-stained recipient cells increased with prolonged co-culture time, suggesting that the internalization of exosomes by recipient cells was time-dependent (Fig. 1e). Collectively, these findings provided evidence that hypoxia enhances exosome secretion from CRC cells and that these exosomes can be efficiently internalized by adjacent cancer cells. The exosome secretion and uptake experiments were conducted using HCT116 cells as a representative cell line, as they exhibited the most significant increase in exosome secretion under hypoxia.

### Hypoxia promotes the exosomal packaging and release of miR-1225-5p in CRC cells

**Figure 2:**
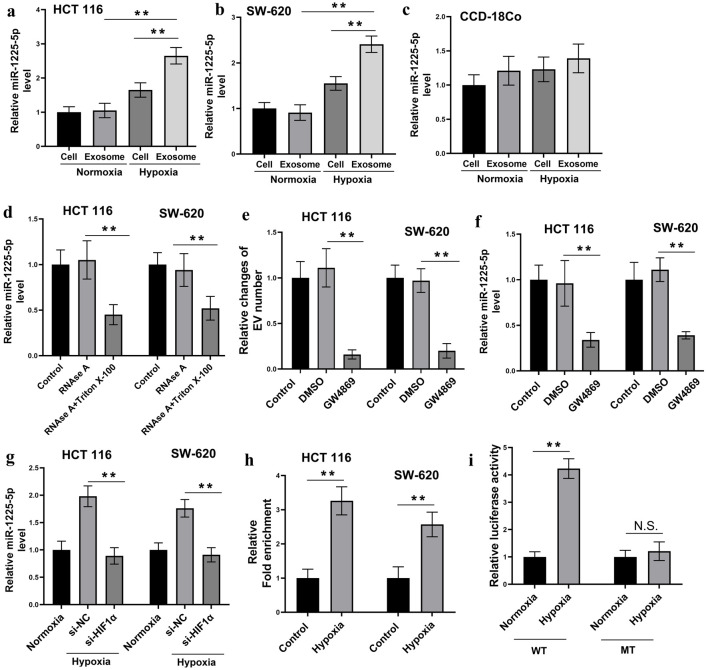
Hypoxia promotes exosomal secretion of miR-1225-5p in CRC cells. (a–c) Relative expression levels of miR-1225-5p in HCT116 (a), SW620 (b) and CCD-18Co (c) cells and their exosomes under normoxic and hypoxic conditions, measured by qRT-PCR. (d) Conditioned media from HCT116 and SW620 cells were treated with RNase A or RNase A + Triton X-100. The results of the QRT-PCR analysis showed that miR-1225-5p levels were not affected by RNase A alone, but were significantly decreased by RNase A + Triton X-100. (e) Relative changes of EV numbers in CM after CRC cells were treated with the exosome secretion inhibitor GW4869 or vehicle control (DMSO). (f) CRC cells were treated with GW4869 or DMSO. The qRT-PCR analysis revealed reduced miR-1225-5p levels in CM after GW4869 treatment. (g) The levels of miR-1225-5p after siRNA was used to knock down the expression of HIF-1α under hypoxia condition. (h, i) The direct transcriptional regulation via HIF-1α binding to the miR-1225-5p promoter was verified by CHIP assay (h) and the luciferase activity assay (i). Data are presented as mean ± SD (n = 3 independent experiments). **P < 0.01 between indicated groups. CRC: colorectal cancer; qRT-PCR: quantitative real-time polymerase chain reaction; CM: conditioned medium; DMSO: dimethyl sulfoxide; siRNA: small interfering RNA; HIF-1α: hypoxia-inducible factor 1-alpha; ChIP: chromatin immunoprecipitation; NC: negative control.

Accumulating evidence indicates that exosome release by tumor cells is tightly regulated by hypoxia, with particular regard to miRNAs [22–24]. In order to explore the potential regulatory miRNAs involved in hypoxia-induced CRC exosome communication, miRNA levels were examined in HCT116 and SW620 cell lysates and their corresponding exosomes cultured under normoxic and hypoxic conditions. The results displayed that hypoxia significantly increased levels of miR-1225-5p in both cells and their respective exosomal fractions, with a more pronounced elevation of miR-1225-5p levels in exosomal fractions (Fig. 2a, b). In contrast, in normal colonic epithelial cells (CCD-18Co), hypoxia failed to significantly change the levels of miR-1225-5p in cells or exosomal fractions (Fig. 2c). These findings indicated that in CRC cells, hypoxia not only enhances the cellular expression of miR-1225-5p, but also promotes the selective packaging of miR-1225-5p into exosomal fractions.

To determine whether extracellular miR-1225-5p is indeed protected within exosomes rather than being freely released, we treated CRC cell-CM with RNase A alone or in conjunction with the detergent Triton X-100. Treatment with RNase A alone did not result in a noticeable change in miR-1225-5p levels in CM, indicating that the miRNA was protected from enzymatic degradation. Conversely, the combination of RNase A and Triton X-100, a detergent that disrupts the exosomal membrane, led to a substantial decrease in miR-1225-5p levels (Fig. 2d). These findings collectively indicated that the majority of miR-1225-5p is encapsulated within the exosomes. In addition, treatment with the exosome secretion inhibitor GW4869 at 10 µM resulted in a considerable decrease of EV numbers as confirmed by NTA (P < 0.01) and miR-1225-5p levels in the CM of both HCT116 and SW620 cells (Fig. 2e, f), supporting the conclusion that exosomes are the primary carriers of extracellular miR-1225-5p in CRC cells.

To investigate the mechanism underlying hypoxia-induced upregulation of miR-1225-5p, we employed small interfering RNA (siRNA) to knock down the expression of HIF-1α under hypoxic conditions, followed by quantification of miR-1225-5p levels (Fig. 2g). The results demonstrated that transfection with HIF-1α-targeting siRNA (si-HIF-1α) significantly reduced miR-1225-5p abundance (P < 0.01). These findings indicate that hypoxia-driven upregulation of miR-1225-5p is dependent on HIF-1α. The direct transcriptional regulation via HIF-1α binding to the miR-1225-5p promoter was verified by CHIP assay (Fig. 2h) and the luciferase activity assay (Fig. 2i). ChIP assay showed that HIF-1α was significantly enriched in the miR-1225-5p promoter region under hypoxia compared to normoxia (P < 0.01). Overall, these results highlighted that hypoxia enhances both the expression and exosomal packaging of miR-1225-5p in CRC cells, with the miRNA predominantly enclosed within exosomes instead of existing in a free extracellular form.

### Exosomal miR-1225-5p is transferred into recipient CRC cells and induces aggressiveness

**Figure 3:**
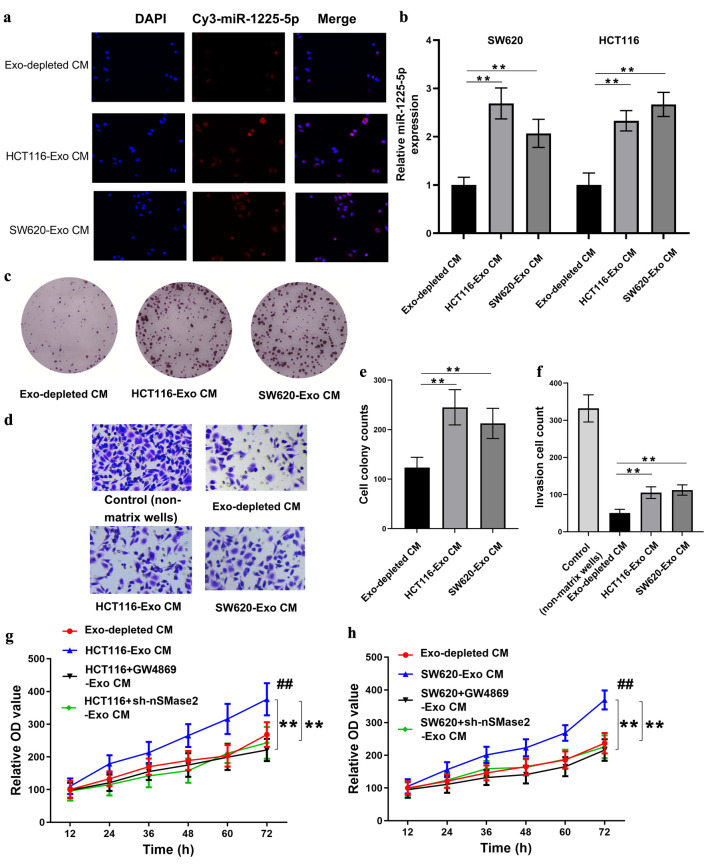
Exosomal miR-1225-5p is transferred into recipient CRC cells and induces cell aggressiveness. (a) Fluorescence microscopy of HCT116 cells after co-culture with Cy3-labeled miR-1225-5p exosomes derived from HCT116 or SW620 cells. Red fluorescence indicated miR-1225-5p transfer. (b) The intracellular miR-1225-5p levels in CRC cells after treatment with exosome-depleted CM or exosomes-containing CM from HCT116 or SW620 cells. (c, e) Colony formation assay and quantification showing increased proliferation after exosomal treatment. (d, f) Transwell invasion assay and quantification showing enhanced invasive capacity in recipient cells exposed to exosomes-containing CM. (g, h) Cell viability was assessed by CCK-8 assay to evaluate the effect of GW4869 and sh-nSMase2. **P < 0.01 between indicated groups. Data are presented as mean ± SD (n = 3 independent experiments). ^##^P < 0.01 compared to Exo-depleted CM. CRC: colorectal cancer; Exo: exosomes; Exo-CM: exosome-containing conditioned medium; Exo-depleted CM: exosome-depleted conditioned medium; CCK-8: cell counting kit-8; sh: short hairpin RNA; NC: negative control.

In an attempt to determine whether CRC-derived exosomal miR-1225-5p could be delivered to recipient cells, Cy3-labeled miR-1225-5p was incorporated into exosomes from SW620 and HCT116 cells and incubated with HCT116 cells. Fluorescence microscopy revealed a strong Cy3 signal (red) in the cytoplasm of HCT116 cells after co-culture with the labeled exosomes, indicating successful transfer of the miRNA via exosomal delivery (Fig. 3a). Subsequently, HCT116 and SW620 cells were exposed to CM (Exo-CM) comprising exosome preparations from HCT116 or SW620 cells. It was observed that the intracellular levels of miR-1225-5p were significantly elevated in both cell lines following treatment with Exo-CM, thereby substantiating the efficacious transfer of exosomal miRNAs (Fig. 3b).

The functional impact of exosomal miR-1225-5p on CRC cells was the focus of further investigation, with the proliferation and invasion of these cells being evaluated. Colony formation assays confirmed that treatment with HCT116-Exo CM or SW620-Exo CM significantly enhanced the clonogenic capacity of recipient HCT116 cells (Fig. 3c, e). Concurrently, Transwell invasion assays revealed a significant increase in invasive cell numbers following exposure to exosomes-enriched CM (Fig. 3d, f). These observations support the notion that exosomal miR-1225-5p augments the aggressive capabilities of CRC cells. Furthermore, the cell proliferation ability was significantly increased by the hypoxia-treated HCT116-Exo CM or SW620-Exo CM, but significantly decreased by treatment with Exo-CM derived from cells treated with GW4869 or sh-nSMase2 (Fig. 3g, h; P < 0.05).

### *CPM* is suppressed in CRC and correlates with patient prognosis

**Figure 4:**
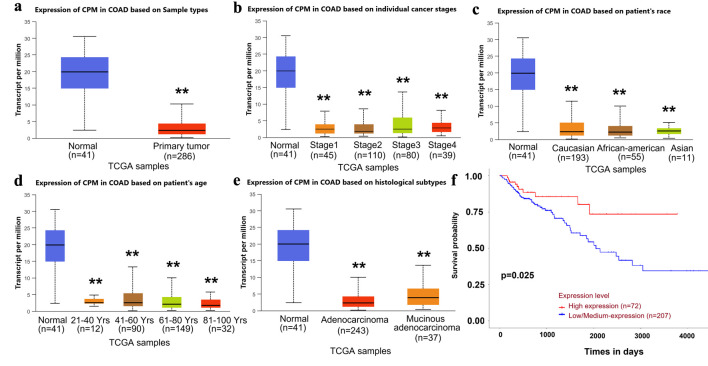
Bioinformatic analysis reveals low *CPM* expression in CRC and its association with poor prognosis. (a) *CPM* expression in normal compared with primary tumor samples (TCGA-COAD dataset). (b) *CPM* expression stratified by tumor stage. (c) *CPM* expression based on patient race. (d) *CPM* expression in different age groups. (e) *CPM* expression across histological subtypes. (f) Kaplan–Meier curves comparing overall survival in CRC patients with high and low/medium *CPM* expression levels. Data are presented as mean ± SD. **P < 0.01 between indicated groups. CRC: colorectal cancer; CPM: carboxypeptidase M; TCGA-COAD: The Cancer Genome Atlas-Colon Adenocarcinoma.

The analysis of the TCGA dataset via the UALCAN online platform identified that *CPM* expression was markedly reduced in primary CRC tumor tissues (Fig. 4a). Further stratification revealed that *CPM* expression was low in all pathological stages (stages I–IV) compared to normal controls (Fig. 4b), suggesting that downregulation of *CPM* is a common feature throughout CRC progression. Subgroup analyses based on patient race and age showed similar trends, with *CPM* expression notably suppressed in all race and age groups (Fig. 4c, d). Furthermore, *CPM* expression was found to be reduced in both adenocarcinoma and mucinous adenocarcinoma subtypes of CRC (Fig. 4e). The survival analysis demonstrated a strong association between low *CPM* expression and poor overall survival. Patients exhibiting high *CPM* expression exhibited a marked improvement in survival (P = 0.0025; Fig. 4f), suggesting that CPM may serve as a favorable prognostic biomarker in CRC.

### Knockdown of *CPM* facilitates CRC cell aggressiveness

**Figure 5:**
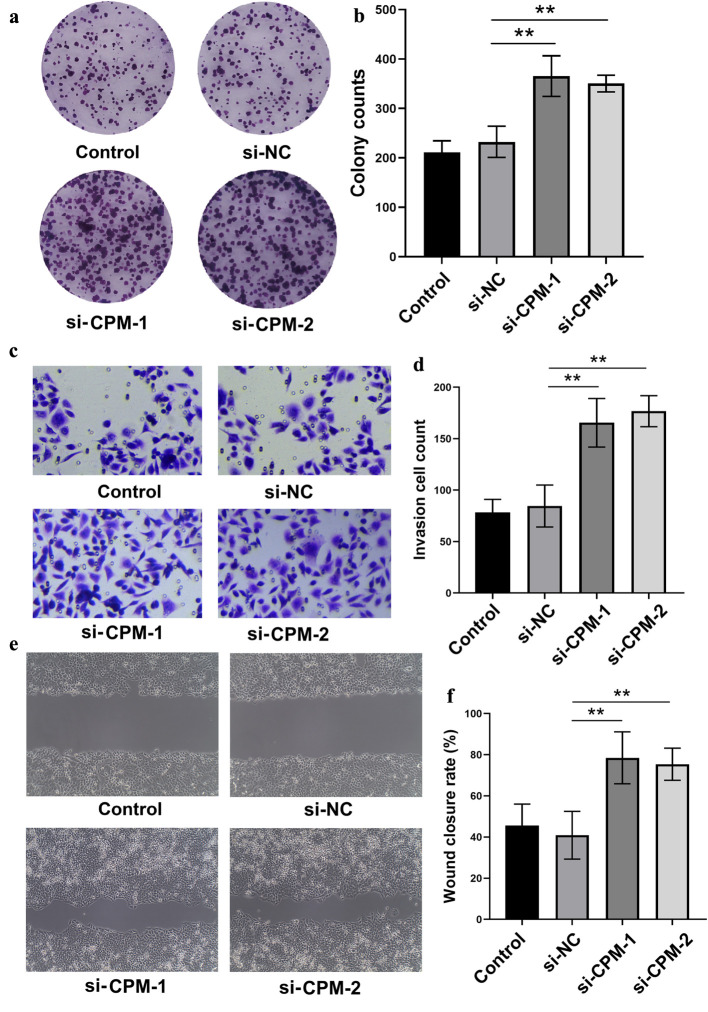
Knockdown of *CPM* facilitates CRC cell aggressiveness. (a, b) Colony formation assay and quantification in SW620 cells after transfection with si-*CPM* or control siRNA. (c, d) Transwell invasion assay showing increased invasive cell counts upon *CPM* knockdown. (e, f) Wound healing assay showing enhanced cell migration following *CPM* silencing. **P < 0.01 between indicated groups. CRC: colorectal cancer; CPM: carboxypeptidase M; siRNA/si: small interfering RNA; NC: negative control; KD: knockdown.

Subsequent investigation was then conducted into the functional relevance of CPM in tumor progression. *CPM* was silenced in SW620 CRC cells using two independent siRNAs, and it was found that a considerable enhancement of colony forming ability was observed in the knockdown group (Fig. 5a, b). The Transwell invasion assay further illustrated that inhibition of *CPM* expression resulted in a markedly increased number of invading cells (Fig. 5c, d). Wound healing assays revealed that cells with reduced *CPM* levels exhibited faster migration, as indicated by a higher wound closure rate (Fig. 5e, f). In conclusion, these experiments supported a tumor suppressor activity of CPM in CRC, as its downregulation enhanced cell proliferation, invasion, and motility.

### MiR-1225-5p facilitates CRC cell proliferation by targeting *CPM*

**Figure 6:**
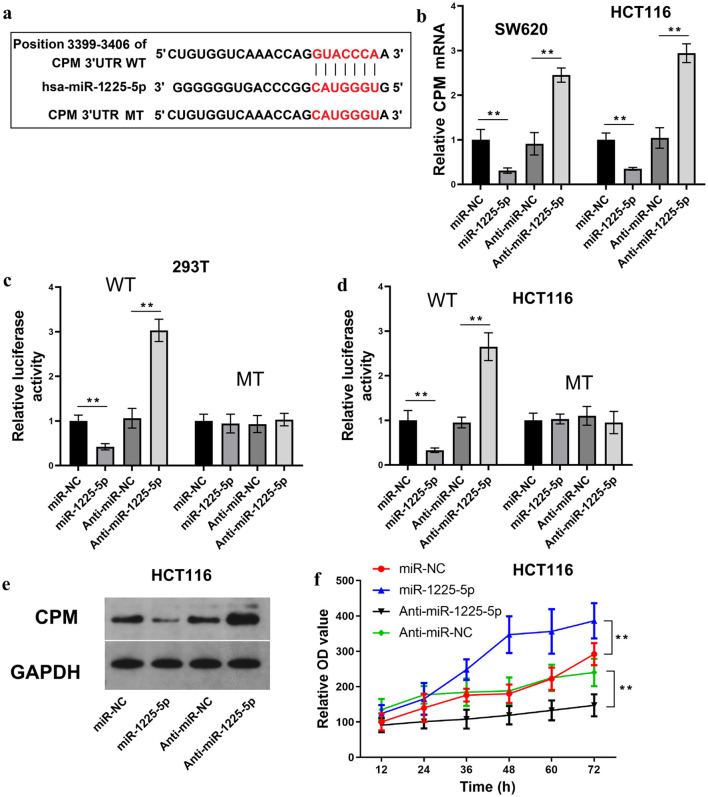
MiR-1225-5p facilitates CRC cell proliferation by directly targeting *CPM*. (a) Predicted binding site of miR-1225-5p in the *CPM* 3'UTR and the sequence of the mutant construct. (b) Relative *CPM* mRNA levels in SW620 and HCT116 cells transfected with miR-1225-5p mimic or inhibitor. (c, d) Luciferase reporter assays in 293T and HCT116 cells co-transfected with miR-1225-5p mimic or inhibitor and WT or MT *CPM* 3'UTR constructs. (e) Protein levels of CPM in HCT116 cells after miR-1225-5p modulation. (f) CCK-8 proliferation assays in HCT116 cells transfected with miR-1225-5p mimic or inhibitor. Data are presented as mean ± SD (n = 3 independent experiments). **P < 0.01 between indicated groups. CRC: colorectal cancer; CPM: carboxypeptidase M; UTR: untranslated region; WT: wild-type; MT: mutant; CCK-8: cell counting kit-8; NC: negative control.

Given the observed tumor-suppressive properties of CPM and its downregulation in CRC, we next investigated whether *CPM* is a direct target of miR-1225-5p. *In silico* analysis revealed a potential miR-1225-5p binding site located in the 3'UTR of *CPM* mRNA (Fig. 6a). In validation of this interaction, we generated wild-type (WT) and mutant (MT) constructs of the *CPM* 3'UTR for use in luciferase reporter assays. The results of qRT-PCR analysis indicated that overexpression of miR-1225-5p markedly decreased *CPM* mRNA levels in both SW620 and HCT116 cells, whereas inhibition of miR-1225-5p exerted an opposing effect (Fig. 6b). In a consistent finding, luciferase activity was severely suppressed in 293T (Fig. 6c) and HCT116 (Fig. 6d) cells co-transfected with miR-1225-5p and the WT *CPM* 3'UTR reporter, while anti-miR-1225-5p restored luciferase activity. These effects were abolished when using the mutant (MT) construct, confirming a direct targeting relationship. In line with these findings, WB analysis confirmed a corresponding reduction in CPM protein levels upon miR-1225-5p overexpression in HCT116 cells, whereas anti-miR-1225-5p restored *CPM* expression (Fig. 6e). Functionally, CCK-8 assays revealed that miR-1225-5p overexpression significantly promoted HCT116 cell proliferation, whereas miR-1225-5p inhibition suppressed cell growth (Fig. 6f). Consequently, these results established *CPM* as a direct downstream target of miR-1225-5p and suggested that miR-1225-5p facilitates CRC cell proliferation by repressing *CPM* expression.

### Exosomal miR-1225-5p promotes CRC cell malignancy by suppressing *CPM*

To validate that exosomal miR-1225-5p regulates *CPM* expression and promotes tumor progression, we collected exosomes derived from hypoxia-treated SW620 cells previously transfected with either miR-1225-5p mimics or inhibitors. These exosome samples were then applied to SW620 and HCT116 recipient cells.

**Figure 7:**
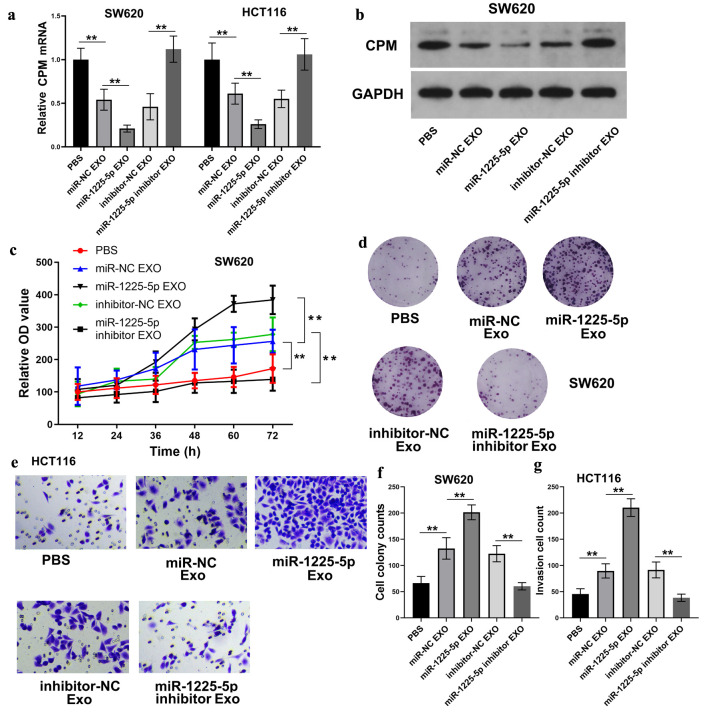
Exosomal miR-1225-5p promotes CRC cell malignancy via *CPM* suppression. (a) *CPM* mRNA levels in CRC cells exposed to exosomes from hypoxia-conditioned SW620 cells that were transfected with miR-1225-5p mimic or inhibitor. (b) Protein levels of CPM in SW620 cells treated with corresponding exosomes. (c) CCK-8 assays showing SW620 cell proliferation after exosome treatment. (d, f) Colony formation assays and quantification in SW620 cells. (e, g) Transwell invasion assays and quantification in HCT116 cells. Data are presented as mean ± SD (n = 3 independent experiments). **P < 0.01 between indicated groups. CRC: colorectal cancer; CPM: carboxypeptidase M; CCK-8: cell counting kit-8; Exo: exosomes; CM: conditioned medium; NC: negative control.

qRT-PCR analysis revealed a profound downregulation of *CPM* mRNA levels by miR-1225-5p-enriched exosomes, whereas exosomes containing miR-1225-5p inhibitors restored *CPM* expression (Fig. 7a). Consistent with this, CPM protein levels were also diminished by miR-1225-5p-rich exosomes and increased by miR-1225-5p-depleted exosomes (Fig. 7b). Functionally, exosomes carrying miR-1225-5p significantly promoted CRC cell proliferation as evidenced by increased cell viability (Fig. 7c) and colony formation (Fig. 7d–f). Conversely, the growth and clonogenic potential of CRC cells were suppressed by exosomes derived from cells transfected with a miR-1225-5p inhibitor. Moreover, Transwell assays evidenced that miR-1225-5p-enriched exosomes augmented the invasive capacity of recipient HCT116 cells, whereas miR-1225-5p-depleted exosomes exerted the opposite effect (Fig. 7e–g). These results represented strong functional evidence that exosomal miR-1225-5p promotes CRC progression by downregulating *CPM* and promoting proliferation and invasion.

### *In vivo* experiments reveal the role of exosome-derived miR-1225-5p and CPM in tumor growth

**Figure 8:**
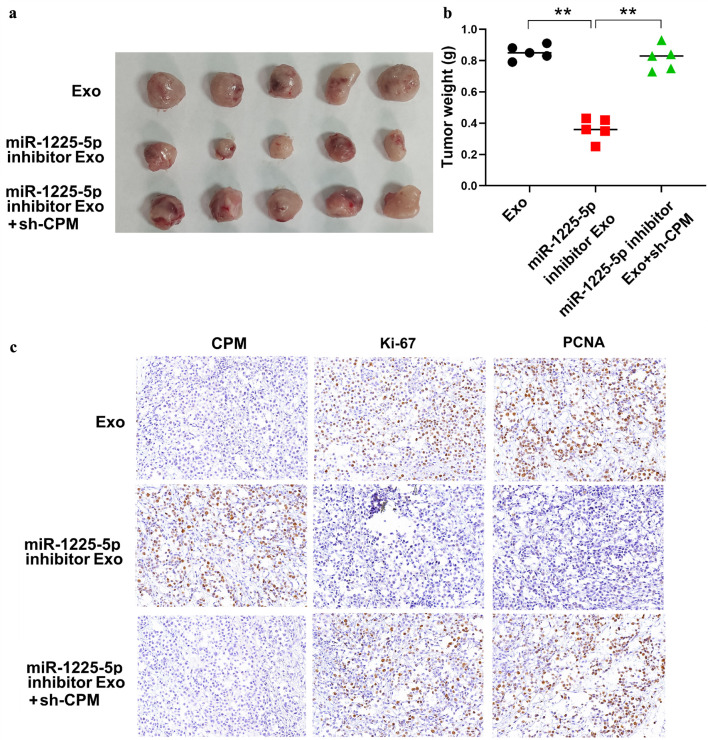
*In vivo* experiments revealed the role of exosome-derived miR-1225-5p and CPM in tumor growth. (a) Images of tumors from three groups in tumor xenograft experiments. (b) The result of tumor weight measurement. (c) Representative images of the IHC assays of CPM, Ki-67, and PCNA in tumor tissue in tumor xenograft experiments. Exo: the tumors were implanted with normal SW620 cells and injected with exosomes from hypoxia-exposed SW620 cells; miR-1225-5p inhibitor Exo: the tumors were implanted with normal SW620 cells and injected with exosomes from hypoxia-exposed SW620 cells transfected with a miR-1225-5p inhibitor; miR-1225-5p inhibitor Exo+sh-CPM: the tumors were implanted with SW620 cells pretreated with sh-CPM and injected with exosomes from hypoxia-exposed SW620 cells co-transfected with the miR-1225-5p inhibitor. Data are presented as mean ± SD (n = 5 mice per group). **P < 0.01 between indicated groups. IHC: immunohistochemistry; CPM: carboxypeptidase M; PCNA: proliferating cell nuclear antigen; Exo: exosomes; sh: short hairpin RNA; NC: negative control; KD: knockdown.

To validate the role of exosome-derived miR-1225-5p and CPM in tumor growth, tumor xenograft experiments were performed using BALB/c nude mice. The miR-1225-5p inhibitor was utilized in rescue experiments to verify the specificity of the axis. By inhibiting miR-1225-5p in hypoxia-treated SW620 cells, we aimed to demonstrate whether the suppression of CPM and the subsequent aggressive phenotypes could be reversed. Exosomes derived exclusively from cells transfected with miR-1225-5p inhibitor were intratumorally injected into mice. The recipient tumors were formed by subcutaneous implantation of SW620 cells, which had been stably transfected with sh-CPM prior to the exosome injection. The tumor size and weight results demonstrated that exosomes isolated from hypoxia-exposed SW620 cells transfected with a miR-1225-5p inhibitor significantly attenuated the tumor-promoting effects compared to the control exosomes, while subcutaneous implantation of SW620 cells pre-transfected with sh-CPM abolished the effect of miR-1225-5p inhibitor (Fig. 8a, b).

Furthermore, the IHC assays showed that exosomes isolated from hypoxia-exposed SW620 cells transfected with a miR-1225-5p inhibitor significantly increased the expression of CPM in tumor tissue, but decreased the expression of Ki-67 and PCNA (Fig. 8c). In contrast, subcutaneous implantation of SW620 cells pre-transfected with sh-CPM significantly increased the expression of Ki-67 and PCNA, counteracting the effect of miR-1225-5p inhibitor (Fig. 8c). These results confirmed that miR-1225-5p delivered from exosomes of hypoxia-exposed SW620 cells promoted tumor growth by inhibiting CPM expression *in vivo*.

## Discussion

As a major type of solid tumor, CRC contributes significantly to the global cancer burden. The complex and heterogeneous TME, in which hypoxia acts as a universal and critical driver, poses a significant challenge for effective clinical management [1, 35–37]. Hypoxia not only triggers metabolic reprogramming but also profoundly alters intercellular communication, primarily through the enhanced secretion of extracellular vesicles such as exosomes [38–40]. In this study, we elucidated a novel intercellular communication network within the hypoxic TME of CRC. We demonstrated that hypoxia stimulates the exosomal packaging and secretion of miR-1225-5p. Upon internalization by neighboring CRC cells, exosomal miR-1225-5p accelerates tumor proliferation, migration, and invasion by directly targeting and suppressing the tumor suppressor gene CPM. To the best of our knowledge, this is the first study to establish the hypoxia/HIF-1α/exosomal miR-1225-5p/CPM regulatory axis in CRC.

Understanding the upstream regulatory mechanisms of exosomal cargo loading under stress is crucial. Our study provides definitive mechanistic insights by demonstrating that hypoxia-driven upregulation of miR-1225-5p is directly orchestrated by HIF-1α. Through rigorous ChIP and luciferase reporter assays, we confirmed that HIF-1α transcriptionally activates miR-1225-5p by binding to its promoter region. Furthermore, RNase protection assays and exosome release inhibition (via GW4869) confirmed that this miRNA is predominantly safeguarded and disseminated via exosomes rather than as free molecules. This elucidates a complete “hypoxia/HIF-1α/exosomal miRNA” cascade, shedding light on how hypoxic CRC cells actively sculpt their microenvironment to favor disease progression.

While previous studies have hinted at the presence of exosomal miR-1225-5p in other malignancies, its specific functions in CRC remained undefined. For instance, in ovarian cancer, hypoxia-induced exosomal miR-1225-5p was shown to drive M2 macrophage polarization via TLR2 signaling [26]. In gastric cancer, its elevated levels in peritoneal lavage fluid were linked to peritoneal dissemination [27]. Our findings significantly expand the functional repertoire of miR-1225-5p. To our knowledge, this is the first study to establish miR-1225-5p as a direct oncogenic mediator of tumor-to-tumor cell communication in CRC. By transferring from hypoxic cores to normoxic or less aggressive regions, exosomal miR-1225-5p propagates aggressive phenotypes across the tumor bulk.

Crucially, our study identified CPM as the direct downstream functional target of miR-1225-5p. CPM, historically recognized as a diagnostic biomarker co-amplified in certain liposarcomas [28–31], exhibited a different role in our CRC models. The contrasting roles of CPM, acting as an amplified oncogene in mesenchymal liposarcomas while functioning as a tumor suppressor in epithelial colorectal carcinomas, highlight a fascinating tissue-specific functional dichotomy. We hypothesize that this dual role may heavily depend on distinct epigenetic landscapes, the availability of cell-type-specific co-factors, or divergent downstream signaling networks inherent to mesenchymal versus epithelial lineages.

Our bioinformatic analysis using TCGA datasets compellingly revealed that CPM expression is significantly suppressed across all pathological stages of CRC, and lower CPM levels strongly correlate with poorer overall survival. In line with these clinical observations, our *in vitro* functional assays confirmed that silencing CPM independently triggers enhanced CRC cell clonogenicity and invasiveness. This establishes CPM as a robust tumor suppressor gene in the context of CRC. This nature of CPM is consistent with a previous study by Lu et al [32], which reported that intracellular miR-146a promotes CRC cell migration by directly targeting CPM. However, our study significantly advances this paradigm. While Lu et al focused on canonical intracellular miRNA regulation, our research introduces a completely different upstream environmental driver: hypoxia-induced, HIF-1α-mediated transcription. More importantly, we highlight the critical role of exosomal packaging for intercellular communication within the TME. By utilizing exosomal miR-1225-5p as a delivery vehicle, we demonstrate a unique paracrine mechanism whereby hypoxic CRC cells can remotely silence this protective CPM gene in adjacent recipient cells, thereby releasing the brakes on tumor progression and propagating aggressive phenotypes across the tumor bulk.

Exosome-mediated delivery of miR-1225-5p effectively silences this protective gene, thereby releasing the brakes on tumor progression. The functional relevance of this regulatory axis was strongly corroborated by our *in vivo* xenograft models. Intercepting this communication network by treating tumors with exosomes derived from miR-1225-5p inhibitor-transfected cells significantly retarded tumor growth and restored CPM expression *in vivo*. Furthermore, this tumor-suppressive effect was successfully reversed upon concurrent knockdown of CPM, perfectly mirroring our *in vitro* mechanistic findings. These *in vivo* data not only validate the pathological significance of the HIF-1α/miR-1225-5p/CPM axis but also highlight the therapeutic potential of targeting exosomal miRNA delivery.

Despite the robust evidence presented, this study has certain limitations that warrant further investigation. Specifically, while our *in vivo* xenograft experiments successfully validated the oncogenic role of the exosome-derived miR-1225-5p/CPM axis, these assays were conducted using immunodeficient BALB/c nude mice. Consequently, this model inherently lacks a fully functional adaptive immune system, precluding the observation of complex interactions with tumor-infiltrating lymphocytes (TILs) within the TME.

The TME is a highly dynamic ecosystem where exosomes mediate profound crosstalk not only between cancer cells but also between cancer cells and immune populations. How hypoxia-induced exosomal miR-1225-5p interacts with TILs remains a critical unanswered question. Exosomal miRNAs are well-documented to facilitate immune evasion by promoting CD8^+^ T cell exhaustion or expanding immunosuppressive regulatory T cells (Tregs). Furthermore, as previously noted in ovarian cancer, hypoxia-induced exosomal miR-1225-5p can drive M2 macrophage polarization [26], which classically suppresses TIL activation and fosters an immunosuppressive niche. Additionally, given that the target gene CPM is a membrane-bound protease capable of altering the local peptide microenvironment (e.g., kinin peptides) [30], its suppression by miR-1225-5p might further alter the recruitment and functional state of TILs. Therefore, we hypothesize that in an immunocompetent setting, exosomal miR-1225-5p might exacerbate CRC aggressiveness not merely through direct oncogenic effects on adjacent cancer cells, but also by blunting TIL-mediated anti-tumor immunity. Future investigations utilizing immunocompetent syngeneic mouse models are urgently warranted to fully elucidate the impact of the exosomal miR-1225-5p/CPM axis on TIL dynamics and immune evasion. Furthermore, the diagnostic potential of exosomal miR-1225-5p as a circulating biomarker needs to be validated in blood or stool samples from large cohorts of CRC patients.

In conclusion, the present findings establish a novel paradigm in which hypoxia-driven, HIF-1α-mediated transcription promotes the exosomal transfer of miR-1225-5p, which consequently suppresses the protective CPM gene and drives CRC malignancy. Our study maps a comprehensive “hypoxia/HIF-1α/exosome/miR-1225-5p/CPM” regulatory network. Targeting this axis, either by inhibiting specific exosome secretion pathways or by restoring CPM function, may offer promising new therapeutic vulnerabilities for combating advanced CRC.

## Supplementary Material

Suppl 1 Primer sequences and annealing temperatures used for qRT-PCR.

## Data Availability

The datasets generated and/or analyzed during the course of this study are accessible from the corresponding author.
