# Supplementary material for: Hypoxia-Induced Exosomal miR-1225-5p Accelerates Colorectal Cancer Progression by Targeting Carboxypeptidase M
Source: World J Oncol. 2026 Jun 25;17(4):477–93. doi: 10.14740/wjon2766 (PMC13375411; doi:10.14740/wjon2766)
Supplement: Suppl 1 — Primer sequences and annealing temperatures used for qRT-PCR. [file wjon-17-04-477-s001.docx]

**Suppl 1. Primer sequences and annealing temperatures used for qRT-PCR.**

| **Gene / miRNA** | **Forward Primer Sequence (5’ → 3’)** | **Reverse Primer Sequence (5’ → 3’)** | **Annealing Temperature (°C)** |
| --- | --- | --- | --- |
| CPM | CAGGAAGGGATGGAAGCGTT | GTGTTCCTTTGGAAACCGCC | 60 |
| HIF-1α | GAACGTCGAAAAGAAAAGTCTCG | CCTTATCAAGATGCGAACTCACA | 60 |
| GAPDH | CCACTCCTCCACCTTTGAC | ACCCTGTTGCTGTAGCCA | 60 |
| miR-1225-5p | GGGTACGGCCCAGTG | GAACATGTCTGCGTATCTC | 60 |
| U6 | CTTGGCAGCACATATACT | AAAATATGGAACGCTTCACG | 60 |

Note: GAPDH was used as the endogenous control for mRNA normalization, and U6 was utilized as the endogenous control for miRNA normalization.
